# Supplementary material for: Implementation Activities in Smoke-Free Public Housing: The Massachusetts Experience
Source: Int J Environ Res Public Health. 2022 Dec 21;20(1):78. doi: 10.3390/ijerph20010078 (PMC9819479; doi:10.3390/ijerph20010078)
Supplement: Supplementary file 1 [file ijerph-20-00078-s001.zip › ijerph-1983127-supplementary.pdf]

Supplementary Materials

Table S1. Descriptive statistics of public housing authorities with smoke-free policy prior to January 2018 (n=161)

	n (%)
Rural	42 (26.1)
Having family housing units on the property (vs. units designated only for elderly/disabled residents)	135 (84)
Federally funded (vs. state funded)	66 (41)
Size (total units)	
1-50	16 (10)
51-250	92 (57)
251-500	24 (15)
501-1000	15 (9)
>1000	14 (9)
Time since the smoke-free policy implementation	Mean: 3.0, SD: 2.1

Note. There were missing values on time since the policy implementation (n=5, 3%) and funding source (n=1, 0.6%).

Table S2. Marginal probabilities of practicing implementation strategies by four groups of public housing identified from latent class analysis (n=161)

	Group 1 (n=61, 38%)	Group 2 (n=24, 15%)	Group 3 (n=39, 24%)	Group 4 (n=37, 23%)	Group 5 (n=37, 23%)
Information sessions for residents	0.72	0.90	0.69	0.93	0.98
Treatment or referral for help with smoking cessation	0.74	0.95	0.95	0.93	0.96
Resident engagement					
Advised where smoking is allowed	0.36	0.90	0.50	0	0.96
Advised on enforcement process	0.08	1.00	0.26	0.04	0.97
Educate or guidance for fellow residents	0.03	0.49	0	0.04	0.69
Helped survey residents about support for the policy	0.02	0.75	0	0.04	0.39
Advised on inclusion/exclusion of e-cig	0.02	0.74	0.06	0	0.65
Staff training					
Health effects of secondhand smoke	0	0	0.17	0.89	0.88
General information about the policy	0.06	0.05	0.63	1.00	1.00
Advice for residents on smoking cessation	0	0	0.22	1.00	1.00
Resources for helping smokers quit	0	0.06	0.37	0.96	1.00
Communication, negotiation skills	0	0	0	0.72	0.65
Resident outreach and engagement	0	0	0.07	0.79	0.73
Procedures for identifying violations	0	0	0.65	1.00	0.86
Procedures for responding to violations	0	0	0.70	1.00	0.86
Partnerships with outside community org.	0.43	0.51	0.84	0.82	0.75
Use of a toolkit	0.27	0.06	0.59	0.57	0.64
Outdoor smoking area	0.52	0.40	0.54	0.29	0.64

Table S3. Survey Questions

1. Before the smoke-free rule was put in place, did the housing authority conduct a survey to get residents opinions about it?

2. Did your housing authority host management-led information sessions for residents about the policy?
3. Which of the following activities took place before or after the smoke-free rule was in effect? Check all that apply.

	Before smoke-free rule in effect	After smoke-free rule in effect
Offered residents referrals to off-site smoking cessation counseling		
Offered on-site smoking cessation counseling or classes		
Offered residents on-site smoking cessation medications (for example patches, gum, lozenges)		
Offered suggestions to contact primary care provider about smoking cessation		
Offered smoking cessation information (Quitline number, booklet, brochure, etc.)		
Other (please specify):		
None of the above		

4. Has the housing authority (or resident groups) developed partnerships with any of the following community organizations to plan or implement, or enforce the smoke-free rule? Check all that apply.

- ☐ External Tenants' rights organizations
- ☐ Internal residents' advocacy groups
- ☐ Community health center
- ☐ Local hospitals
- ☐ Local health departments
- ☐ DPH funded technical assistance providers (for example, Chris Banthin/Public Health Advocacy Institute (PHAI) or Kathleen McCabe/Health Resources in Action (HRiA))
- ☐ The Center for Tobacco Treatment Research and Training at UMass Amherst
- ☐ Fire departments

- ☐ Community service agencies
- ☐ Residents' advocacy groups
- ☐ Local judges/courts
- ☐ Voluntary organizations (i.e. American Lung Association)
- ☐ University/academic groups or organizations
- ☐ Other (please specify) ______________________________________

5. Did you utilize any toolkits or materials from HUD or other organizations (for example, How to Go Smoke-Free: A Toolkit for Multiunit Housing) in the implementation of the smoke-free rule?

- ☐ Yes
- ☐ No
- ☐ No tools available
- ☐ Don't know

Resident Inclusion

6. Did residents help decide whether or not to have a smoke-free rule?
   - ☐ Yes
   - ☐ No

7. In what ways did residents help with the smoke-free rule? (check all that apply)

	Residents not involved	Residents contributed *before* smoke-free rule in effect	Residents contributed *after* smoke-free rule in effect
Advised *where* smoking is permitted			
Advised on enforcement processes			
Provided education or guidance to fellow residents (tips for compliance or smoking cessation, storytelling about importance of rule, etc.)			
Helped survey residents about support for the smoke-free rule			
Advised on inclusion/exclusion of e-cigarettes			
Other (please specify)			

8. To the best of your knowledge, what is the level of overall support of the policy among residents now?

- Non-supportive
- Mostly unsupportive
- Somewhat unsupportive
- Neither unsupportive or supportive
- Somewhat supportive
- Mostly supportive
- Nearly all supportive
- Don't know

Staff Training

9. Using the grid below, please indicate whether staff received each kind of training, and if so, which staff got the training. Check all that apply.

	Property Manager	Resident Services Coordinator	Maintenance Staff	Security Staff	Other
Health effects of secondhand smoke exposure					
General information about the smoke-free rule (e.g. where people can and can't smoke)					
Basic advice for residents on smoking cessation					
Resources available for helping smokers quit					
General communication and negotiation skills					
Resident outreach and engagement					
Procedures for identifying violations					
Procedures for responding to violations					
Other (please specify):					

Smoking Regulations

Now we would like to ask you some details on the specifics of your smoke-free rule

10. Does your smoke-free rule *allow* smoking in certain indoor and/or outdoor places on housing authority property?

- ☐ Yes
- ☐ No
- ☐ Don’t know

11. Please select ALL the places on the property where people *are* permitted to smoke:

- ☐ Individual unit balconies
- ☐ Common area patios outdoors
- ☐ Designated outdoor smoking areas (for example a *specific* location, bench, shelter, or gazebo)
- ☐ Designated indoor smoking areas
- ☐ Parking lot
- ☐ Away from building entrances and doorways (for example, beyond a certain distance)
- ☐ In cars parked at the property
- ☐ Other (please specify): ____
- ☐ Smoking is prohibited everywhere on the property
